# Hypertension Health Promotion via Text Messaging at a Community Health Center in South Africa: A Mixed Methods Study

**DOI:** 10.2196/mhealth.4569

**Published:** 2016-03-10

**Authors:** Damian Hacking, Hanne J Haricharan, Kirsty Brittain, Yan Kwan Lau, Tali Cassidy, Marion Heap

**Affiliations:** ^1^ Health and Human Rights Programme Department of Public Health and Family Medicine University of Cape Town Cape Town South Africa

**Keywords:** telemedicine, health knowledge, attitudes, practice, developing countries, hypertension

## Abstract

**Background:**

The use of mobile phones to deliver health care (mHealth) is increasing in popularity due to the high prevalence of mobile phone penetration. This is seen in developing countries, where mHealth may be particularly useful in overcoming traditional access barriers. Non-communicable diseases may be particularly amenable to mHealth interventions, and hypertension is one with an escalating burden in the developing world.

**Objective:**

The objective of this study was to test whether the dissemination of health information via a short message service (SMS) led to improvements in health knowledge and self-reported health-related behaviors.

**Methods:**

A mixed methods study was carried out among a cohort of 223 hypertension clinic patients, in a resource-poor setting in Cape Town, South Africa, in 2012. Hypertensive outpatients were recruited at the clinic and administered a baseline questionnaire to establish existing knowledge of hypertension. Participants were then randomly assigned to intervention or control groups. The intervention group received 90 SMSes over a period of 17 weeks. Thereafter, the baseline questionnaire was readministered to both groups to gauge if any improvements in health knowledge had occurred. Those who received SMSes were asked additional questions about health-related behavior changes. A focus group was then conducted to obtain in-depth feedback about participants’ experience with, and response to, the SMS campaign.

**Results:**

No statistically significant changes in overall health knowledge were observed between the control and intervention groups. The intervention group had positive increases in self-reported behavior changes. These were reaffirmed by the focus groups, which also revealed a strong preference for the SMS campaign and the belief that the SMSes acted as a reminder to change, as opposed to providing new information.

**Conclusions:**

Although the content of the SMSes was not new, and did not improve health knowledge, SMSes were effective in motivating positive self-reported behavior change among hypertensive patients.

**Trial Registration:**

Pan African Clinical Trials Registry Number: PACTR201412000968462. Registered 18 December 2014 (Archived by WebCite at http://www.webcitation.org/6fhtyLRcO).

## Introduction

mHealth, the use of mobile phones to support health care, is often seen to have great potential, as mobile technology has a high prevalence, with 6.8 billion subscriptions worldwide [1]. This extends to the developing world, with Africa having the fastest growing market for cell phone technology, and over 75% of the low-income South African population already owning, or having access to, a cell phone number [2]. mHealth can be implemented for a variety of health-related functions: to provide information, act as reminders of clinic visits, encourage behavior changes, and be actively used in symptom diagnoses and drug adherence checking [3][4][5]. However, there are few evaluations that thoroughly investigate the viability of the mHealth intervention, typically drawing on either quantitative or qualitative data, but not both. Furthermore, cost-effectiveness, context, and acceptability are still contentious issues, and therefore, even though strong potential exists, the viability of mHealth interventions in all situations is not clear and further investigation into such matters is still merited [6][7].

The use of mHealth interventions in the control and management of non-communicable diseases is relatively well established; however, there is a paucity of research on its viability from lower-middle-income countries [8]. Hypertension is one of the many non-communicable diseases on the rise in the developing world, including South Africa [9], where the prevalence is approximately 21% [10]. This can in part be attributed to increasing rates of rural-urban migration, as hypertension is relatively uncommon in rural South Africa [11]. It is further exacerbated by socioeconomic factors that encourage poor diet and sedentary lifestyles and contribute to low knowledge levels (both of risk factors and treatment) amongst the poorer urban population, which ultimately increases complications in hypertension [12]. Low knowledge levels in hypertensive patients are one of the key barriers to hypertensive care [13]. Furthermore, there is a particular demand for lifestyle modification information in South Africa [14], and treatment and control of non-communicable diseases are part of South Africa’s National Development Plan [15].

mHealth interventions represent an opportunity to overcome the significant access barriers to health care seen in developing countries [16][17], due to the traditional socioeconomic and rural and urban divides [6], as well as an opportunity to address low knowledge levels and contribute to positive behavior change. According to social cognitive theory, short message service (SMS) interventions may be particularly good at increasing knowledge, as they allow for repeated learning, grant a high level of autonomous learning, and allow the learner to learn at their own pace and in a low-stress environment [18]. This paper therefore seeks to test whether the dissemination of health information via SMS leads to improvements in health knowledge and self-reported health-related behaviors amongst chronic hypertensive patients in a resource-poor setting.

## Methods

An SMS campaign consisting of both knowledge and lifestyle information was designed. A mixed methods approach that employed a controlled clinical trial and a focus group evaluated the SMS campaign. The project was approved by the University of Cape Town’s Health Sciences Faculty Human Research Ethics Committee (HREC REF 043/2011).

The details of this mixed methods approach are described below.

### Recruitment and Study Setting

Participants (n=223) were recruited from a chronic hypertension outpatient clinic support club at a community health center in the Gugulethu township of Cape Town. Gugulethu is a densely populated poor urban settlement with 95.5% of dwellers speaking Xhosa as their mother tongue, 35% unemployment with an average yearly household income of US $3300 (2001), and the majority falling within an age range of 20-34 years [17]. Participants were assigned to experiment (n=109) or control groups (n=114) alternating in order of recruitment. To ensure informed consent, fieldworkers used an information sheet and consent form to brief participants about the project in their language of choice—either English or Xhosa. All participants signed consent forms both at recruitment and prior to the exit interview. The possibility of being in the control group was detailed for the participants in the consent form. The intervention group received the SMSes, and the control group received standard of care and no SMSes. All participant data were stored safely either in a locked cupboard or on a secure, password-protected cloud server.

### Text Messaging

SMSes were designed in conjunction with health promoters and staff at the Gugulethu Community Health Centre, and reviewed by academics and clinicians from the Department of Family Medicine at the University of Cape Town. The SMSes were then translated from English into Xhosa (2 of the 3 official languages of the Western Cape, the provincial site of the study) and back translated to ensure that the meaning of the message was accurately preserved. A total of 90 SMSes were created and disseminated over 17 weeks (see Multimedia Appendix 1) and included 2 introductory SMSes and reminders of the option to opt out. Information in the SMSes covered knowledge of hypertension and healthy lifestyle suggestions. “Did you know” introduced the knowledge SMSes and “Health Tips,” the lifestyle SMSes.

### Knowledge and Self-Reported Behavior Change Assessment

Control and intervention arm participants were administered a preintervention multiple-choice questionnaire (26 questions). The questionnaire explored demographic profiles (7 questions) and baseline knowledge of symptoms, risk factors, health behavior, and how to control hypertension (19 questions) (see Multimedia Appendix 2).

After the intervention, the same questionnaire was administered to participants in both the control and intervention groups, with additional behavior-related questions administered to the intervention group (see Multimedia Appendix 3). The scoring was as follows: for the questions that had 1 correct answer, a score of 1 was assigned if the participant chose the correct answer, and a score of −1 if they chose the incorrect answer or a score of 0 if they answered that they did not know. For the questions where participants could give more than 1 answer, the number of incorrect answers was subtracted from the number of correct answers. Further details on scoring are outlined in Multimedia Appendix 4.

### Focus Group

In order to further explore the results from the questionnaire, 7 participants who received SMSes partook in a 2-hour focus group, held at the community health center. Participants were chosen at random and invited via phone call until a total of 10 had confirmed, although only 7 arrived on the day. The focus group was conducted by 1 investigator, observed by 1 research assistant, and recorded using a Dictaphone and note taking. A language translator was present at the focus group, allowing participants to communicate in the language of their choice. The topics explored knowledge change, the impact and efficacy of SMSes, and the relationship between SMS receival and behavior change, and are outlined in the focus group discussion guide (Multimedia Appendix 5). Recordings were transcribed and thematic analysis was carried out in consultation with the investigator’s and researcher’s notes.

### Statistical Analysis

Fisher’s exact tests were performed to determine statistical difference between the control and intervention groups for all questions, except for the questions that had multiple correct answers, in which case, 2 sample *t* tests were performed. Statistical significance was set at *p*<.05.

## Results

### Participants

**Figure 1:**
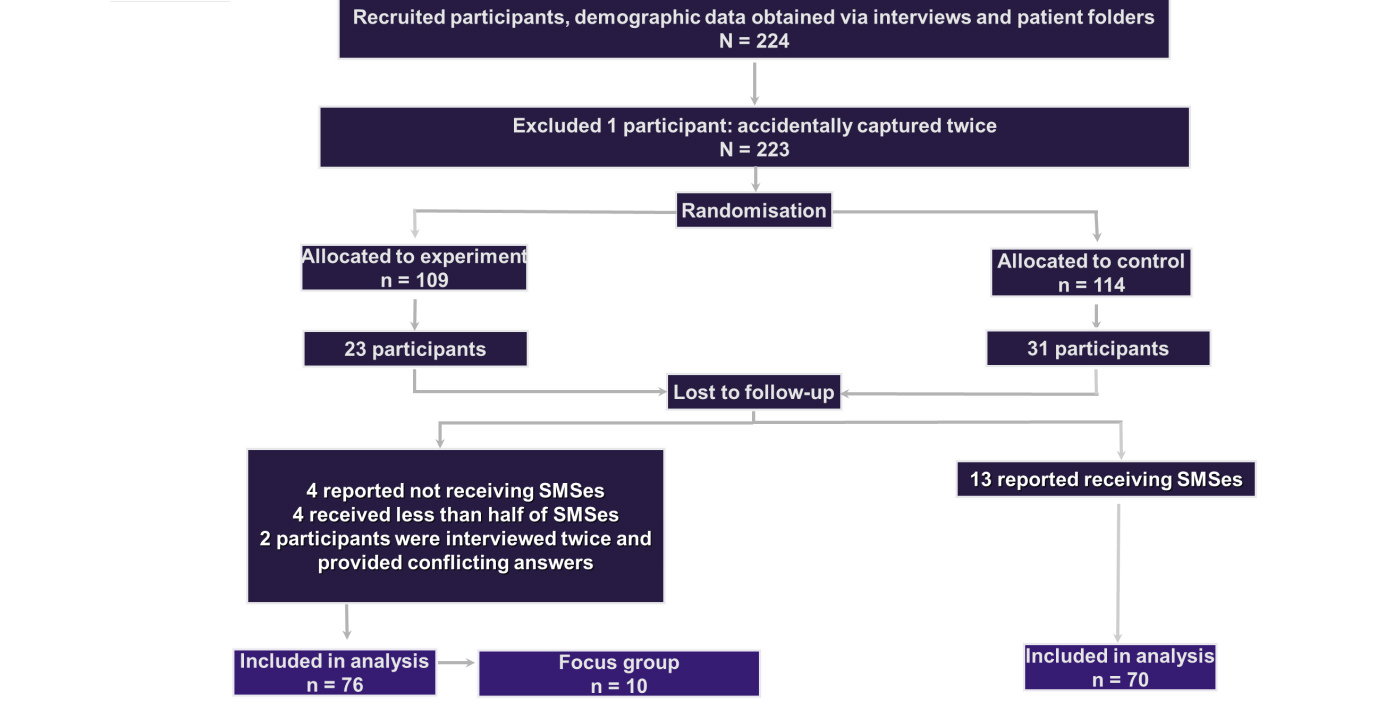
Participant diagram. 224 participants were originally recruited, but due to loss to follow up and various exclusion criteria, only 146 (76 in experimental arm, 70 in control arm) were included in the final analysis.

In total, 224 participants were recruited (Figure 1). Of the 224 participants, 1 was a duplicate and 54 (24%) were lost to follow-up between baseline and exit interview (23 in the intervention arm and 31 in the control arm); a further 10 in the intervention arm were excluded from analysis for reporting receiving fewer than half the SMSes or being accidentally interviewed in duplicate and giving conflicting answers; while 13 were excluded in the control arm as they reported erroneously receiving SMSes at the exit interview.

The mean age of the cohort was 52.83 years, with a standard deviation of 11.62 years and a range between 26.81 and 85.73 years. Most participants (108/146, 74.0%) were female. In total, 64.4% (94/146) indicated that Xhosa was their first language; 43.8% (64/146) indicated that they were married; the majority indicated that they had some form of secondary education (84/146, 56.9%), with 2/146 (1.4%) indicating that they had a tertiary qualification; just under half (67/146, 45.9%) indicated that they were unemployed; less than a third indicated that they were employed (40/146, 27.4%); and 26.0% (38/146) indicated that they received a pension. A fifth (29/146, 19.9%) indicated that they had no monthly income; similarly, just less than a third (43/146, 29.5%) indicated that their monthly income was less than US $500.

No problematic demographic differences were observed between the control and intervention groups at baseline. In terms of baseline knowledge scores, both groups had an average total of 17 out of 31 (54.8%) (Table 1).

**Table 1:** Comparison of baseline demographics and knowledge scores between control and intervention groups.

		Intervention	Control	Overall sample
Age – Mean (range)		54.23 (27.9-86.19)	54.44 (26.81-92.15)	54.34 (26.81-92.15)
Gender – N (%)				
	Female	79 (77.45%)	85 (82.52%)	164 (80.0%)
	Male	23 (22.55%)	18 (17.48%)	41 (20.0%)
Language – N (%)				
	Xhosa	66 (60.55%)	72 (63.16%)	138 (61.88%)
	English	43 (39.45%)	40 (35.09%)	83 (37.22%)
	Afrikaans	0 (0.0%)	2 (1.75%)	2 (0.90%)
Marital status – N (%)				
	Married	48 (44.04%)	47 (41.23%)	95 (42.60%)
	Single living with partner	5 (4.59%)	3 (2.63%)	8 (3.59%)
	Single living with family	47 (43.12%)	51 (44.74%)	98 (43.95%)
	Single living with others	2 (1.83%)	2 (1.75%)	4 (1.79%)
	Divorced	3 (2.75%)	1 (0.88%)	4 (1.79%)
	Widow or widower	4 (3.67%)	10 (8.77%)	14 (6.28%)
Education – N (%)				
	Below Grade 7	20 (18.35%)	26 (22.81%)	46 (20.63%)
	Between Grade 7 and 12	63 (57.80%)	62 (54.39%)	125 (56.05%)
	Passed matric	23 (21.10%)	24 (21.05%)	47 (21.08%)
	No matric but diploma	2 (1.83%)	1 (0.88%)	3 (1.35%)
	Postmatric qualification	1 (0.92%)	1 (0.88%)	2 (0.90%)
Employment status – N (%)				
	Unemployed	55 (50.46%)	48 (42.11%)	103 (46.19%)
	Employed	26 (23.85%)	32 (28.07%)	58 (26.01%)
	Pension	27 (24.77%)	34 (29.82%)	61 (27.35%)
	Not looking for employment	1 (0.92%)	0 (0.0%)	1 (0.45%)
Monthly income – N (%)				
	None	24 (22.02%)	18 (15.79%)	42 (18.83%)
	Less than R4000	26 (23.85%)	32 (28.07%)	58 (26.01%)
	R4000-R10,000	1 (0.92%)	2 (1.75%)	3 (1.35%)
	Pension or social grant	58 (53.21%)	62 (54.39%)	120 (53.81%)
Hypertensive knowledge score – Mean (range)		8.77 (1-15)	8.54 (2-16)	8.65 (1-16)
Health-seeking behavior score – Median (range)		8 (2-10)	8 (4-11)	8 (2-11)
Overall score – Median (range)		17 (3-24)	16 (8-22)	17 (3-24)

### Knowledge and Self-Reported Behavior Changes

Overall, no significant changes in knowledge were observed between the control and intervention arms at exit. However, in the individual questions, the intervention arm demonstrated a significantly higher knowledge of what to do in terms of medication adherence when they leave Cape Town, their place of residence, for an extended duration (81% vs 94%, *p*<.05 (see Table 2)). This is an important piece of knowledge, as there is a high seasonal migration of this population from Cape Town to the more rural Eastern Cape province of South Africa during holiday periods.

**Table 2:** Knowledge scores for control and intervention groups at exit.

Question	Control score	Intervention score	*P* value
Condition questions overall score (observed range)	9.71 (5-13)	9.6 (6-15)	.77
-What is high blood pressure? (% correctly answered)	59%	49%	.06
-What is normal blood pressure?	59%	73%	.31
-What puts you at risk for developing high blood pressure? (score)	2.897	2.744	.45
-Does everybody who has high blood pressure have symptoms?	56%	62%	.20
-What complications may you prevent by managing your high blood pressure?	1.176	1.103	.67
-Can you stop taking your medication without asking your doctor?	97%	92%	.38
-Can high blood pressure be cured?	87%	87%	.79
-When you leave Cape Town, which one of the following is right?	81%	94%	.03
-If you have high blood pressure, contact the clinic if you have which of the following symptoms?	1.25	1.19	.68
Behavior questions overall score (observed range)	7.78 (5-10)	8.05 (6-10)	.15
-Can you reduce your blood pressure?	91%	94%	.89
-Is smoking good or bad if you have high blood pressure?	99%	99%	>.99
-Is keeping a normal weight important if you have high blood pressure?	99%	96%	.62
-How much should you drink, if at all, if you have high blood pressure?	51%	56%	.64
-How much should you exercise 3 days a week?	29%	28%	.12
-What are the best ways to manage your stress?	1.221	1.372	.14
-Do eating habits affect blood pressure?	74%	85%	.07
-Is salt good or bad for a person with high blood pressure?	18%	12%	.54
-What should you cut down on if you have high blood pressure?	99%	99%	>.99
-Are vegetables and fruit good for a person with high blood pressure?	99%	100%	.22
Overall score (observed range)	17.5 (11-23)	17.7 (13-23)	.69

Positive self-reported behavior change was reported by participants in the SMS intervention for all categories questioned (see Table 3).

**Table 3:** Self-reported behavior changes for intervention arm.

Behavior change	Self-reported answer (%)			
	Yes	No	Don’t know	N/A
Have you stopped smoking since the SMS campaign?	13	0	1	86
Have you lost weight since the SMS campaign?	58	28	5	9
Have you reduced your alcohol intake since the SMS campaign?	12	6	3	79
Have you increased your exercise since the SMS campaign?	81	12	1	6
Have you started eating healthier since the SMS campaign?	88	5	3	4
Have you reduced your consumption of red meat since the SMS campaign?	65	9	8	18
Have you increased your consumption of fruit and vegetables since the SMS campaign?	87	1	1	10
Have you reduced your salt intake since the SMS campaign?	63	3	10	24

### Focus Group Results

All participants in the focus group reiterated the self-reported behavior change results. Thematic analysis found that the SMSes were generally viewed in a positive light, with 4 themes being elucidated. Firstly, the SMSes were viewed not as a source of new information, but rather as a motivation for change, which was felt to be extremely useful:

Ok, for me since I hear this thing time and again, wherever I go, I go to Groote Schuur [hospital], I go to Victoria hospital, even here and see dieticians, wherever I go, I am told these things, but I never took them seriously. But the SMSes made me believe in all the things that I‘ve been told all the time, and made me took the matters on my own hands. You know, like I took them seriously, and do everything. Because sometimes I do get pamphlets to read and read them and realise that they are related to everything that the SMSes are telling you what to do, but the SMSes made me took matters into my own hands, and do things right.

Many participants thus suggested that the SMSes functioned as a reminder to change, which emerged as the second theme:

I did know before, but a lot because it [the SMSes] always come and remind me so I was, always my mind was always pressured on, so it was good for me.

As I received the SMSes, especially where they were telling me on what to eat, and what to reduce, on like salt, and then about what kind of fat, margarine or soft butter you must use, I have changed, everything according to what the SMSes were telling me. And, ever since I got on this project I have managed to lose a little bit of weight.

Thirdly, when the participants were asked why the SMSes motivated them, they described the SMSes as “trustworthy” and “caring” and felt like they were being looked after:

because those people [researchers] that took our particulars were from the University of Cape Town, they were very educated people. They are like doctors to us, you know; got a lot of information on how to keep our health, keep uh, healthy.

Lastly, they also stressed that SMSes were readily available compared with other forms of information; participants felt that having the information on their mobile phone was more useful than getting a pamphlet or getting information from health professionals:

I did get the information from the dietician, but I did not follow up. When you are getting the SMSes it’s a reminder, it’s on your phone. You can always read your messages, it always reflects. So it is easy for you to follow-up than being told full scale listening to someone.

It’s between you and your phone, there’s no one next to you, so by reading you are thinking back of your mind ‘I think I will try to do this one’.

## Discussion

The results demonstrated almost no differences in knowledge between the control and intervention arms at exit, with the exception of 1 question. Self-reported behavior change in the intervention group showed significant trends toward healthier lifestyles. This observation was confirmed by focus groups, which emphasized the utility of the SMSes as a reminder, and a source of motivation to change, rather than a source of new knowledge.

There are a few possible explanations for no observed difference in knowledge results between the control and intervention groups. Firstly, the survey questions may not have been suitable in assessing participants’ knowledge; they were not sufficiently difficult for measuring a change. Census data on baseline education levels for our population demographic indicate the majority have not finished secondary schooling [17], and this informed the design of our survey questions. Furthermore, baseline questions were formulated in conjunction with health care professionals around what information was felt was important for the patient to know. While low levels of education were confirmed by our baseline assessment demographic data, it appears participants’ specific health knowledge behavior was relatively high. Indeed, they may have been so high (participants knew on average 54.8% of the information based on their baseline questionnaires) that there may not have been enough leeway for any statistically significant gains to be observed. In many ways, this is a positive, as it suggests that the health care staff are providing good health information, among other possible sources. This may also be due to the fact that our patient population was sampled from a hypertensive club, which may not be reflective of general patients with hypertension. Subgroup analysis stratified by some of the baseline demographics, such as education level, may have accounted for such factors but could not be performed due to limited sample size. Secondly, methodological issues were encountered with the SMSes service delivery. On average, 29.7% of SMSes failed to deliver to the intervention group. This was due to the service provider and casts doubt on the validity of the results. Lastly, loss to follow-up was also a significant problem, which reduced the statistical power of the results.

The findings do nonetheless suggest that SMSes are effective in motivating behavior change. However, there is a high potential for bias, as only *self-reported* behavior changes were reported. The health behavior change results could not be confirmed objectively with blood pressure and weight changes before and after the intervention, due to poor medical record keeping in patient clinic folders. While the focus group findings supported the self-reported behavior change findings from the questionnaire, the same self-reporting biases exist, and there may also be selection bias at play in the focus group, as participants self-selected into the focus group. The focus group further suggested that the SMSes were functioning as “cues to action”—see, for example, the Health Belief Model [19]. The Health Belief Model theorizes that health seeking behavior is influenced by a range of beliefs, such as perceived severity, perceived benefits, self-efficacy (confidence in one’s ability to take action), as well as reminders to action that activate the readiness of the patient to seek good health. The SMSes may act as one such reminder, as they provide information as well as remind the patient of this information on a daily basis. Furthermore, the Stages of Change theory [20] postulates that behavior change is a staged process starting at precontemplation and working through 5 stages toward maintenance or termination of the behavior. The SMS campaign appears to be particularly good at moving from the precontemplation to the contemplation or preparation stage. It is uncertain whether the campaign was long enough or particularly well suited to moving participants into the next stage of “action,” although self-reported behavior change suggests it might have been. Combined, these results suggest an expressed interest in changing behavior following the SMS intervention, even if it is uncertain whether this resulted in behavior change.

In conclusion, the results suggest that more research is required before implementing SMS interventions for chronic disease in developing countries, and that any intervention should be entered with caution, realizing some of the limitations of the developing world. Firstly, the infrastructure for their successful implementation may not be available [21], a problem experienced in this intervention by a high SMS delivery failure rate. Secondly, they may not be a necessary, or good, vehicle for knowledge dissemination. However, SMSes do appear to act as a reminder for the patient, and participants reported they felt they were being better looked after by receiving the SMSes, and that the SMSes were cueing positive health-related behavior changes. This could potentially make them a very useful tool for assisting in the management of chronic conditions. Future studies should incorporate objective measures of behavior change, such as weight and blood pressure changes. Recent trials have also suggested that personalization of the SMSes and tailoring toward the individual receiving the SMS can have a marked improvement on outcomes, a theme that could also be explored in future work [22].

## Appendix

Multimedia Appendix 1 List of hypertension SMS campaign SMSes.

Multimedia Appendix 2 Hypertension SMS campaign baseline questionnaire.

Multimedia Appendix 3 Hypertension SMS campaign exit questionnaire.

Multimedia Appendix 4 Questionnaire scoring calculations.

Multimedia Appendix 5 Focus group discussion guide.
