# Supplementary material for: Hypertension Health Promotion via Text Messaging at a Community Health Center in South Africa: A Mixed Methods Study
Source: JMIR Mhealth Uhealth. 2016 Mar 10;4(1):e22. doi: 10.2196/mhealth.4569 (PMC4807246; doi:10.2196/mhealth.4569)
Supplement: Multimedia Appendix 1 [file mhealth_v4i1e22_app1.pdf]

Multimedia Appendix 1

Hypertension SMS campaign

SMSes

Welcome. You'll receive 5 SMSs per week for 16 weeks. We hope we can help with good health information so you can lower your blood pressure.

Greetings: You can recognise SMSs from us, as they will start with 'health tips' and 'did you know'. We will use BP as short for 'blood pressure'.

Health tip: Let’s get started. A good way to lower BP is to exercise.

Did you know? If you do not want to continue receiving BP SMSs, send a 'please call me' to 0793212612

Did you know? High blood pressure is the force/pressure of blood flowing through the arteries in your body.

Did you know? Normal blood pressure is said to be 120/80. Remember to ask your health care provider if you have any questions or need more information.

Health tip: There are many things you can do to lower your BP. Make the right choice today. Stop smoking if you smoke. Smoking increases blood pressure.

Did you know? Blood pressure which is above 130/80 puts you in danger of having high blood pressure

Health tip: Know your BP. It is important. Ask your health professional at your next visit to the clinic.

A health tip: To reduce blood pressure, eat food which has less salt, do not eat too much red meat and sausages

Health tip: Your health is important. Write down your BP every time you visit the clinic to see if the changes you make are helping

Did you know? Eating good food helps you to control your blood pressure. Watch for SMSs which have helpful tips

Health tip: If you are overweight, lose weight to lower your blood pressure. Start today, but remember it will take time

Did you know? High BP can’t be cured. But a healthy lifestyle can help you manage it. Look out for health tips.

Did you know? Looking after yourself can help manage your BP. Follow our tips on how to change your lifestyle.

Health tip: Make simple healthy lifestyle changes: Eat more fish, skinless chicken and vegetables. Eat less red meat and less salt.

Did you know? High blood pressure is a chronic disease. That means you will have it forever.

A health tip: How are you? To be mentally exhausted and pressurised can increase your blood pressure.

Health tip: Lower your stress by exercising or talking about your problems. If you can manage your stress, you can lower your BP.

Did you know? Smoking, drinking alcohol, eating unhealthy food, little exercise and being mentally stressed puts you in danger of high blood pressure.

Did you know? Do not forget to question your caregiver about anything or when you need further explanation.

A health tip: Reduce fat from meat before you cook it.

Did you know? Contact a doctor/clinic if you have symptoms, such as pain in chest, stomach cramps or persistent headaches.

Health tip: Remember: Cut down on red meat and salt if you have high blood pressure.

Did you know? If you do not want to continue receiving BP SMSs, send a 'please call me' to 0793212612

Health tip: Make the right choice today to lower your BP. Stop smoking if you smoke. Smoking increases blood pressure.

Did you know? You may have no symptoms and still have high blood pressure. Remember to check your BP regularly.

Did you know? Remember: no symptoms, does not mean that you do not have high blood pressure. Don’t skip visits to the clinic.

Health tip: How are you feeling about having high BP? Remember start exercising today to help lower your BP
.
Health tip: Remember NEVER to change your medication or stop taking your medication unless your doctor tells you to.

Health tip: Remember to go to the clinic to get medication for the time you will be away when you leave Cape Town.

Health tip: Do not share your medication with friends and family. Ask your health care provider if you hae any questions or need more information.

A health tip: Take little salt to reduce your blood pressure.

Did you know? Don’t stop taking your medication because you feel better. Remember you can have high BP without having symptoms.

Did you know? If you have high BP, you need to see a doctor regularly to manage your condition. Don’t miss appointments.

Health tip: Look out for more tips on how to lower BP: manage your stress, lose weight if you are overweight, change eating habits.

Did you know? Keeping a recommended healthy weight is important if you have high blood pressure.

A health tip: A lot can be done to reduce your blood pressure.

Health tip: Do you know whether you weigh more then you should? Get weighed at the clinic or at a pharmacy. Ask about your ideal weight.

A health tip: Control your BP by using medication, visiting your doctor, your caregiver & by changing your lifestyle.

Health tip: Manage your BP by taking medicine, seeing your health care provider regularly, and changing your life style.

Health tip: Want to lower your BP? Stop smoking and drinking alcohol, eat healthily, exercise, maintain recommended weight, and reduce stress.

Health tip: Contact your doctor if you have chest pain, stomach cramps, persistent headaches, shortness of breath, swollen legs.

Did you know? You can lower risk of illness. Take medicine, attend clinic appointments, change lifestyle. Keep up the good

work

Health tip: Want to change, but don't know how? Exercise and changing your eating habits can help you lose weight and lower BP.

Did you know? If you do not want to continue receiving BP SMSs, send a 'please call me' to 0793212612

Did you know?: Changing the way you live can help control your BP. Change is difficult and takes time. Start by changing one thing today.

Health tip: Make one change today to lower your BP: Exercise, stop smoking, manage stress, lose weight or change what you eat.

A health tip: To change your lifestyle can help you control your blood pressure. Look out for health tips

Health tip: Be active: Exercise is one of the best ways to lower your stress. Walk, exercise at home or join a sports club.

A health tip: Try to walk fast, try to exercise at home or at the gymnasium.

Health tip: Did you exercise this week? Aim at exercising 30 minutes at least three times a week.

Health tip: Alcohol can increase your blood pressure. Stop drinking or reduce your drinking to lower your BP.

A health tip: If you are a female, you <u>may</u> take one drink of alcohol per day. If you are a man you <u>may</u> take two drinks of something intoxicating per day.

Health tip: Remember one drink means one beer OR one tot of spirit OR one glass of wine OR one small glass of sherry.

A health tip: If you are a female, you <u>may</u> take one drink of alcohol per day. If you are a man you <u>may</u> take two drinks of something intoxicating per day.

A health tip: Would you like to improve your health? Try this: exercise, stop smoking, stop or reduce alcohol

Did you know? Smoking is bad if you have high blood pressure. Stop smoking to reduce BP. Ask for help to quit.

Health tip: Cook with little salt . Taste food before you add salt. Try it today.

Health tip: Stress can cause high blood pressure. You can reduce stress by exercising. The more the better!

Health tip: Manage stress by exercising and talking about your problems. Talk to your clinic sister or a friend if you need help.

A health tip: Do you have difficulty in changing your lifestyle? Seek help from your clinic nurse. Decide to make a change, one each day.

A health tip: Healthy food can reduce BP. It is also good to reduce weight and keep your body weight healthy.
Health tip: Eat more vegetables, fruit, beans and lentils. All these help reduce high blood pressure.

Health tip: Cook with little salt . Try to cook vegetables with no salt or fat (that is oil or margarine or butter)

Health tip: Cut down on the fat you eat. Too much fat is bad for your blood pressure. Especially fat from animals (meat, milk, eggs).

Health tip: What's for supper tonight? Remember it is better to cook with oil or Flora/Rama margarine than butter.

Health tip: don't eat too many fatty food and deep fried foods such as pies, sausages and chips.

Health tip: Fruit and vegetables are good if you have high BP. Try and eat 5 portions of fruit and vegetables per day.

A health tip: Put vegetables on your rice, pasta, stew and curry.

Health tip: Feeling like snacking? Try a raw carrot, some tomatoes or cucumbers or a fruit. These are healthier than chips and cakes.

Did you know? If you do not want to continue receiving BP SMSs, send a 'please call me' to 0793212612

Health tip: Ready-made and take-away food (like KFC) is often high in fat and salt. Cook your own healthy meals.

Health tip: Use soft tub margarine instead of hard brick margarine.

Health tip: Boil, grill and steam rather than fry. It is a good way

to cut down on fat.

Did you know? Reducing salt intake can reduce your BP. Remember, salt will raise your blood pressure.

Health tip: Cook with little salt and don't add salt to your food. This will help lower your BP.

A health tip: Would you like to keep the taste of food AND reduce salt? Try lemon juice, berbs (basil, sage, origanum), chillis, spices (cinamon).

Health tip: Cut down on polony, salami, ham, bacon, sausages, dried and smoked fish. These have too much salt.

Health tip: Remember! Cut down on salty snacks such as chips, peanuts and biscuits.

A health tip: Use a pinch of Aromat, Mamite, and Oxo. They have too much salt.

A health tip: Eat the skinless chicken and fish instead of eating red meat.

Health tip: Next time buy red meat without fat. Also cut all fat from meat to reduce the fat you eat.

Health tip: Do you want an easy way to reduce fat in your diet? Remove chicken skin before cooking. Most of the fat is in the skin.

Health tip: Choose low fat or skim milk and dairy products. This can help reduce your high BP.

A health tip: Powdered milk has too much fat. It is better to use skimmed milk or milk with low fat.

Health tip: Try a small change for a healthy lifestyle. Eat brown bread instead of white. This can help reduce your high BP.

Health tip: How are you doing? Remember a healthy lifestyle can help reduce high blood pressure. Keep up the good work.

A health tip: Please continue to enquire from the healthcare worker on how you can change your lifestyle and stay healthy

Health tips for lower BP are ending. You will not receive smses anymore. But please continue to live a healthy lifestyle. Good luck!
