# Supplementary material for: Hypertension Health Promotion via Text Messaging at a Community Health Center in South Africa: A Mixed Methods Study
Source: JMIR Mhealth Uhealth. 2016 Mar 10;4(1):e22. doi: 10.2196/mhealth.4569 (PMC4807246; doi:10.2196/mhealth.4569)
Supplement: Multimedia Appendix 2 [file mhealth_v4i1e22_app2.pdf]

Multimedia Appendix 2

Hypertension SMS campaign

Baseline questionnaire

1: Consent signed?

a) Yes

2: Identify group

a) Intervention
b) Control

3: About yourself

a) What is your cell nr?.......................
b) What is your folder nr?........................
c) What is your language of choice?

4: What is your marital status?

a) Married
b) Single living with partner
c) Single living with family
d) Single living with people - not family
e) Widow
f) Divorced

5: What is your highest level of schooling/education?

a) Below grade 7/standard 5.
b) Between grade 7/standard 5, and 12/standard 10
c) Passed Matric
d) No matric but diploma
e) Have post-matric qualification.

6: What is your employment status?

a) Employed
b) Unemployed
c) Pension
d) Not looking for employment
e) Never worked

7: Your monthly income?

a) None
b) Pension/social grant
c) Less than R4000
d) Between R4000 and R 10,000
e) More than 10,000

8: What is high blood pressure? Choose one answer.

a) Fat covering the arteries
b) The force of blood flowing through the arteries
c) Too many white blood cells
d) Don't know

9: What is normal blood pressure?

a) 120/80
b) 130/80
c) 90/70
d) 140/100
e) Don't know

10: What puts you at risk of developing high blood pressure?

a) Smoking
b) Drinking
c) Unhealthy diet
d) Too little exercise.
e) Too much exercise.
f) Eating too little
g) Having unprotected sex.

h) Stress.

i) Being overweight.

j) Being underweight.

k) Too little sleep.

l) Don’t know

11: Does everybody who has high blood pressure have symptoms?

a) Yes, you will always have symptoms

b) No, you can have no symptoms and still have high blood pressure

c) Don’t know.

12: What complications may you prevent by managing your high blood pressure?

a) Stroke

b) Cancer

c) HIV

d) Heart attack

e) Epilepsy

f) Kidney damage

g) Diabetes

h) Don’t know

13: Can you stop taking your medication without asking your doctor?

a) Yes

b) No

c) Don’t know

14: Can high blood pressure be cured?

a) Yes

b) No

c) Don’t know

15: When you leave Cape Town, which one of the following is right?

a) Stop taking your medication

b) Go to the clinic to get enough medication for the time you will be away

c) Get medication from a clinic where you are going

d) Don't know

16: If you have high blood pressure, contact the clinic if you have which of the following symptoms?

a) Persistent headache

b) Feel nausea

c) Chest pain

d) Loose stools

e) Stomach cramps

f) Tiredness

g) Ear ache

h) Don't know

17: Can you reduce your blood pressure?

a) No there is nothing you can do

b) Yes, changing your lifestyle helps to control high blood pressure

c) Don't know

18. Is smoking good or bad if you have high blood pressure?

a) Good

b) Bad

c) Neither good/nor bad

d) Don't know

19: Is keeping a normal weight important if you have high blood pressure?

a) Yes

b) No

c) Your weight does not matter as long as you take your medication

d) Don't know

20: How much should you drink, if at all, when you have high blood pressure?

a) Men can drink 3 drinks daily, women 2

b) As much as you like. It does not affect blood pressure

c) Men can drink two, women one
d) Don’t know.

21: How much should you exercise at least three days a week?

a) 2 hours
b) It does not matter
c) 30 minutes
d) 1 hour
e) You should not exercise at all
f) Don’t know

22: What are the best ways to manage your stress?

a) You can’t manage your stress
b) Eat better
c) Exercise
d) Talk about your problems
e) Drink more alcohol

23: Do eating habits affect blood pressure?

a) No, not at all
b) Yes, a healthy diet helps control high blood pressure
c) Very little can be done to control high blood pressure
d) Don’t know

24: Is it better to cook with?

a) Oil/Margarine (Flora or Rama)
b) Butter
c) It does not matter
d) Don’t know

25: Is salt good or bad for a person with high blood pressure?

a) Too much salt is bad
b) People with high blood pressure need more salt
c) People with high blood pressure should have no salt

d) Neither good/nor bad

e) Don’t know

26: What should you cut down on if you have high blood pressure?

a) Fish

b) Red meat

c) Chicken

d) Vegetables

e) Don’t know

27: Are vegetables and fruit good for a person with high blood pressure?

a) Yes

b) No

c) Maybe

d) Don’t know
