# Supplementary material for: Hypertension Health Promotion via Text Messaging at a Community Health Center in South Africa: A Mixed Methods Study
Source: JMIR Mhealth Uhealth. 2016 Mar 10;4(1):e22. doi: 10.2196/mhealth.4569 (PMC4807246; doi:10.2196/mhealth.4569)
Supplement: Multimedia Appendix 3 [file mhealth_v4i1e22_app3.pdf]

<u>Multimedia Appendix 3</u>

Hypertension SMS campaign

Exit questionnaire

1. Consent signed?

a) Yes

2. What is your folder nr?.......................

3. What is high blood pressure? Choose one answer.

a) Fat covering the arteries
b) The force of blood flowing through the arteries
c) Too many white blood cells
d) Don’t know

4. What is normal blood pressure?

a) 120/80
b) 130/80
c) 90/70
d) 140/100
e) Don’t know

5. What puts you at risk of developing high blood pressure?

a) Smoking
b) Drinking
c) Unhealthy diet
d) Too little exercise.
e) Too much exercise.
f) Eating too little
g) Having unprotected sex.
h) Stress.
i) Being overweight.
j) Being underweight.

k) Too little sleep.
l) Don’t know

6. Does everybody who has high blood pressure have symptoms?

a) Yes, you will always have symptoms
b) No, you can have no symptoms and still have high blood pressure
c) Don’t know.

7. What complications may you prevent by managing your high blood pressure?

a) Stroke
b) Cancer
c) HIV
d) Heart attack
e) Epilepsy
f) Kidney damage
g) Diabetes
h) Don’t know

8: Can you stop taking your medication without asking your doctor?

a) Yes
b) No
c) Don’t know

9: Can high blood pressure be cured?

a) Yes
b) No
c) Don’t know

10. When you leave Cape Town, which one of the following is right?

a) Stop taking your medication
b) Go to the clinic to get enough medication for the time you will be away
c) Get medication from a clinic where you are going
d) Don’t know

11. If you have high blood pressure, contact the clinic if you have which of the following symptoms?

a) Persistent headache
b) Feel nausea
c) Chest pain
d) Loose stools
e) Stomach cramps
f) Tiredness
g) Ear ache
h) Don’t know

12. Can you reduce your blood pressure?

a) No there is nothing you can do
b) Yes, changing your lifestyle helps to control high blood pressure
c) Don’t know

13. Is smoking good or bad if you have high blood pressure?

a) Good
b) Bad
c) Neither good/nor bad
d) Don’t know

14. Is keeping a normal weight important if you have high blood pressure?

a) Yes
b) No
c) Your weight does not matter as long as you take your medication
d) Don’t know

15. How much should you drink, if at all, when you have high blood pressure?

a) Men can drink 3 drinks daily, women 2.
b) As much as you like. It does not affect blood pressure.
c) Men can drink 2, women 1
d) Don’t know.

16. How much should you exercise at least three days a week?

a) 2 hours
b) It does not matter
c) 30 minutes
d) 1 hour
e) You should not exercise at all.
f) Don’t know

17. What are the best ways to manage your stress?

a) You can’t manage your stress
b) Eat better
c) Exercise
d) Talk about your problems
e) Drink more alcohol

18. Do eating habits affect blood pressure?

a) No, not at all
b) Yes, a healthy diet helps control high blood pressure
c) Very little can be done to control high blood pressure
d) Don’t know

19. Is it better to cook with?

a) Oil/Margarine (Flora or Rama)
b) Butter
c) It does not matter
d) Don’t know

20. Is salt good or bad for a person with high blood pressure?

a) Too much salt is bad
b) People with high blood pressure need more salt
c) People with high blood pressure should have no salt
d) Neither good/nor bad
e) Don’t know

21. What should you cut down on if you have high blood pressure?

a) Fish
b) Red meat
c) Chicken
d) Vegetables
e) Don’t know

22. Are vegetables and fruit good for a person with high blood pressure?

a) Yes
b) No
c) Maybe
d) Don’t know

Behaviour change

23. Have you stopped smoking? Choose one answer.

a) Yes
b) No
c) N/A (for non-smokers)

24. Have you lost weight or are you trying to? Choose one answer.

a) Yes
b) No
c) N/A (for people who are not overweight)

25. Have you stopped drinking or reduced your alcohol intake? Choose one answer.

a) Yes
b) No
c) N/A (for non-drinkers)

26. Have you started exercising? Choose one answer.

a) Yes
b) No
c) N/A (for people already physically active)

27. Are you managing your stress better? Choose one answer.

a) Yes
b) No
c) Don't know
d) Don't feel it is important
e) N/A – Does not feel stressed

28. Have you changed your diet? Choose one answer.

a) Yes
b) No
c) N/A (already followed a healthy diet)

29. Have you changed from cooking with butter to oil or Fora/Rama margerine?

a) Yes
b) No
c) N/A (already cooked with oil or rama/flora margarine)

30. Have you cut down on salt in your food?

a) Yes
b) No
c) N/A (already ate little salt)

31. Are you cutting down on red meat? Choose one answer.

a) Yes
b) No
c) N/A (don't eat a lot of red meat)

32. Are you eating more fruit, vegetable, beans and lentils? Choose one answer.

a) Yes
b) No
c) N/A (already eat a lot of these foods)

SMSes

33. Did you receive the SMS?

a) Yes
b) No

34. If yes, you did receive the SMSs – how did you find it

a) Useful
b) A waste of time
c) Don’t know

35. Would you recommend Health Department to introduce the SMS campaign permanently

a) Yes
b) No
c) Not sure
