# Supplementary material for: Hypertension Health Promotion via Text Messaging at a Community Health Center in South Africa: A Mixed Methods Study
Source: JMIR Mhealth Uhealth. 2016 Mar 10;4(1):e22. doi: 10.2196/mhealth.4569 (PMC4807246; doi:10.2196/mhealth.4569)
Supplement: Multimedia Appendix 4 [file mhealth_v4i1e22_app4.pdf]

<u>Multimedia Appendix 4</u>

Score calculation:

Questions specific to the condition of hypertension:

What is high blood pressure?

What is normal blood pressure?

What puts you at risk of developing high blood pressure?

Does everybody who has high blood pressure have symptoms?

What complications may you prevent by managing your high blood pressure?

Can you stop taking your medication without asking your doctor?

Can high blood pressure be cured?

When you leave Cape Town, which one of the following is right?

If you have high blood pressure, contact the clinic if you have which of the following symptoms?

Questions specific to health-seeking behaviour:

Can you reduce your blood pressure?

Is smoking good or bad if you have high blood pressure?

Is keeping a normal weight important if you have high blood pressure?

How much should you drink, if at all, when you have high blood pressure?

How much should you exercise three days a week?

What are the best ways to manage your stress?

Do eating habits affect blood pressure?

Is salt good or bad for a person with high blood pressure?

What should you cut down on if you have high blood pressure?

Are vegetables and fruit good for a person with high blood pressure?

- Note: Question about cooking with butter or margarine excluded, as it was only asked at baseline.
- For the questions which had fixed choice answers, a score of 1 was assigned if the participant chose the correct answer, and a score of -1 if they chose the incorrect answer or a score of 0 if they answered that they did not know.
- For the questions where participants could give more than one answer, the number of incorrect answers was subtracted from the number of correct answers.
