# Supplementary material for: Hypertension Health Promotion via Text Messaging at a Community Health Center in South Africa: A Mixed Methods Study
Source: JMIR Mhealth Uhealth. 2016 Mar 10;4(1):e22. doi: 10.2196/mhealth.4569 (PMC4807246; doi:10.2196/mhealth.4569)
Supplement: Multimedia Appendix 5 [file mhealth_v4i1e22_app5.pdf]

Focus group – guidelines/questions

1. Questions exploring theme 1: Why did knowledge not change significantly?

1. Did you like the sms'es. Why/why not?
2. Do you think other people like them? Why/why not?
3. How many sms'es did you read? Why so few/many?
4. Do you think people read all of them?
5. How did the sms'es make you feel/how did you react when you received them?

   Probe (anticipation, anxiety (especially for high blood), irritation, motivation to change behavior, started to think about changing) Maybe use examples (indicate how people may react. Use examples.
6. Do you know how other people reacted?
7. Did your reaction change over time?
8. Did you understand all the sms'es? Give an example – one of the more difficult sms'es. Probe.
9. Did they sms-campaign give you all the knowledge you felt you need? What were the gaps? What should they have said?

–

Theme 2: Exploring behavior change: Why did they report behavior change (despite no knowledge change)? Did they really change? Did they want to change, did they contemplate changing. Did they feel they had to say they changed (social desirability)?

10. Read out a knowledge sms and a behavior prompting sms – what did you like most? Why? Which one is easiest to remember?

11. How did smses that told you to do something (give an example) – live healthily, not drink, not smoke etc. make you feel? (Irritated? Motivated? Excited? Couldn't be bothered? Anxious – because didn't feel I could do the thing, ambivalence, more easy to remember.)

a. Did you feel that you were back at school? Told what to do? (Did it create resistance?)

b. If you could say that sms'es had a 'voice' whose would it be - 'the sister at the clinic', your mother, your partner, a friend.

c. Did you change your lifestyle bc of sms'es. If yes, what did you change? Was it easy? What made it easy? What made you do it? If no, why not?

d. Did you prefer the sms'es compared to receiving sms'es from the clinic, from pamphlets etc. Why/why not? Why are sms'es better?

e. Did the sms'es make you start thinking about changing behavior/doing something differently? Why/why not?

f. Did some sms'es remind you about things you already knew and motivate you to do something about it? Give examples.

g. Change is not easy and many people tell you to do this and that. Did the sms'es make you feel that you had to change or say you changed even if you felt you could not? Do you think other people would react in that way?

<u>Theme 3: Cell phone use, smses and information sharing</u>

12. Did you share the information from sms'es with anybody? Whom? Why?

a. Did you share with other pregnant women/patients? Women at the clinic? (when sitting next to them, waiting, being bored?)

13. Do you share your cell phone with others (family, partner, friend?). Is cell phone sharing common?

14. Did you change your phone/phone nr? during the sms-campaign?

15. Do you always have you cell phone with you, do you always have it on, charged?

16. Did you keep the sms'es or delete them. Why? Did you read them more than once?

17. How do you feel about the information you received on sms compared to other sources, e.g. the information you received at the clinic. If different, why? (many indicate it is)

18. What would you do to make the sms'es better? (Are there too many, too much repetition, what about the tone, is it polite enough, is it patronizing, demanding). Would you like to receive 'reminders' later?

19. Create your own sms - "how would it sound?" How would you like the 'sms person' to 'speak'.

20. Did you learn anything new from sms'es? What?

21. Something puzzles me: How come so many said they had change behavior, but their knowledge had not changed much. Did you just say that because you had to?
